# MED34/448: The Networked Health-Care Environment of the Future: Requirements for new human abilities

**DOI:** 10.2196/jmir.1.suppl1.e71

**Published:** 1999-09-19

**Authors:** V Patel, E Shortliffe, D Kaufman

**Affiliations:** ^1^McGill University School of MedicineMontrealCanada

**Keywords:** Communication, Internet-based Collaboration, Cognition, Medical Education

## Abstract

The implications of the Internet for health care are increasingly understood as scientists, health workers, patients, and health administrators envision new applications, new means for communicating about health issues, and new ways of accessing pertinent health information at the point of care. It is important to study not only the new technologies themselves, but also to recognize that the optimal use of these technologies requires new skills by users. Not only must both patients and health professionals be taught the basic skills related to use of networking technologies, but those who develop future systems must understand the new human abilities that are implied by the remarkable changes that are envisioned. We describe the results of research that have implications for effectively exploiting networking technology in order to enhance creativity, collaboration, and communication. The development and implementation of enabling tools and methods that provide ready access to knowledge and information are among the central goals of medical informatics. Given the immensity of this challenge, the need for multi-institutional collaboration is increasingly being recognized. Collaboration has typically involved individuals who work together at the same location. With the evolution of electronic communication modalities, workers at Harvard, Columbia, McGill, and Stanford Universities jointly investigated the role that networking technologies can play in supporting research collaboration at a distance. All communications among the workers from the other three institutions were observed in order to gain insights into the limitations and successes of communications technology in supporting this distributed creative process. We analyzed the activities of the Intermed team as they sought to develop a common representation for clinical guidelines, known as the GuideLine Interchange Format (GLIF). These activities can be described as a process of computer-mediated collaborative design. We report here on the cognitive, socio-cultural, and logistical issues encountered when scientists from diverse organizations and backgrounds use communications technologies while designing and implementing shared products. Results demonstrate that the effectiveness of communication modalities is predicated on the specific objectives of the task. We identify suitable uses of email, conference calls, and face-to-face meetings. The leaders play an integral role in guiding and facilitating the group activities across modalities. Most important was the proper use of technology to support the evolution of a shared vision of group goals and methods, an element that is clearly necessary before successful collaborative designs can proceed. We interpret these research findings as they relate to the scientific collaboration via the Internet with specific focus on changes in skills required with these new media of communication.

